# The Myology of the Raven

**Published:** 1891-03

**Authors:** V. C. Vaughan


### REVIEWS.

THE MYOLOGY OF THE RAVEN. A GUIDE TO THE STUDY OF THE MUSCULAR SYSTEM IN BIRDS. By R. W. Shufeldt. Pages, 343. London and New York: Macmillan & Co. 1890.

The student of comparative anatomy, who has desired to acquaint himself with the myology of birds has heretofore been compelled to rely almost exclusively upon the articles by Selenka and Gadow in Bronn's Klassen und Ordnungen des Thier-Reichs and Furbringer's Untersuchungen zur Morphologie und Systematik der Vogel. These, excellent as they are, have possessed the disadvantage to English and American students of being in a foreign language and of being expensive. Therefore there is need of an English book upon this subject, and the writer of the volume before us cannot be accused of inflicting upon us a treatise for the existence of which there is no need. Let us inquire into the manner in which he has attempted to supply the admitted deficiency.

In the first place, what can be said of the author's wisdom in selecting the *corvidae* as the family of birds with which the student can familarize himself with the muscular structure of birds in general? On this point the author writes in his preface as follows: "In choosing the Raven for our subject, it was done in view of the fact that it is a large representative of a very numerous and cosmopolitan family of birds, the corvidae; so that in almost any part of the world, a variety of birds becomes available whose muscular systems can be studied by the aid of the present volume. It is hardly necessary to add that crows of all descriptions, jays, orioles, and a host of others, all fall within this category. It has this advantage too for the teacher and the student at the biological laboratory; for the former can use as his subject the larger and more advantageous specimens, as the ravens or crows, while the latter can confirm the instruction of the former, at home, upon any of the smaller varieties or the corvidae, such as the jays or rooks."

To the above given reasons might be added the fact that in the corvidae the muscles are well developed in proportion to the size of the birds.

The author properly, we think, decides against the adoption of a nomenclature founded upon the nerve supply of the muscle, because, as he states, the same muscle in different types of vertebrates is not always supplied with the same nerve and this would lead to confusion especially to the young student, and moreover, the myology could not then be well studied without, at the same time, a thorough study of the neurology, a task which the student is not always competent to carry out successfully.

In giving an opinion concerning the merits of a book, the following points demand consideration: (1) The accuracy and exactness of the author's statements, (2) The arrangement of the matter, and (3) The suitability of the book to those who are supposed to need and use it. The reviewer has gone through this valuable contribution to comparative anatomy with some care and is glad to say that on the first of the above mentioned points, he has no criticism to offer. Dr. Shufeldt has shown himself master of the subject and deserves the thanks of all naturalists. Indeed, the work is one of which American Scientists have reason to be proud, and it seems out of place to make any criticism. Inaccuracies of statement have been diligently sought for, but with negative results. With full appreciation of the merits of the book the writer begs to offer the following suggestions to the author: The book is likely to be of most service to the independent student. Those pursuing a course of comparative anatomy under competent instructors in our best Universities will have at their command the valuable German works already referred to. This does not imply that to these also the present volume will be of no value. It will be of great service to these, but for them the quotation of page after page in the original German will be unnecessary. It would suffice to give references to Bronn and Eurbringer. To the independent student, however, the value of the book will be greatly enhanced, if the German quotations should in future editions be condensed and their sum and substance given in the English. Indeed, the value of the volume would not in any way be lessened by such a change. If the quotations are to be continued, let them be given in translation. It would be different were our author criticising the German authors. In such a case, the desire to exactly and fairly represent his opponents would justify the giving of the quotations in the original language. But this reason for the production of a polyglot volume does not exist.

A matter of minor importance is the arrangement or order of dissections. The reviewer would recommend his students not to begin with the dermal muscles. While some of these muscles in birds are well developed, others are found with difficulty, or not at all. This fact is well recognized and clearly stated by our author, but we would suggest a footnote advising the independent student to begin with the muscles of the chest and upper extremities and warning him that he must not expect to fully acquaint himself with the anatomy of the raven from the dissection of a single carcass. It is very perplexing and discouraging for the young enthusiastic student to find that the very first muscles which he attempts to study are not recognizable or are present with such variations as to cast grave doubts upon their identity. The dissection of the dermal muscles should be reserved for subsequent study, after the student has acquired the skill, care and judgment which can come only from long continued practical work.

In conclusion the reviewer wishes to reiterate the statement that the volume which he has the honor of introducing to the readers of this journal is worthy of its able, dilligent author, and a credit to the scientific workers of this country.

V. C. VAUGHAN.

